# ROSS SYNDROME WITH ANA POSITIVITY: A CLUE TO POSSIBLE AUTOIMMUNE ORIGIN AND TREATMENT WITH INTRAVENOUS IMMUNOGLOBULIN

**DOI:** 10.4103/0019-5154.70694

**Published:** 2010

**Authors:** Biju Vasudevan, MPS Sawhney, S Vishal

**Affiliations:** *From the Department of Dermatology and Venereology, Base Hospital, Delhi Cantt, India*

**Keywords:** *Ross syndrome*, *autoimmune*, *intravenous immunoglobulin*

## Abstract

A 28-year-old serving soldier presented with patchy areas of absence of sweating and blurring of vision. On examination he was found to have segmental anhidrosis, right sided tonic pupil and absent ankle jerks. Investigations revealed ANA positivity with no other abnormalities. He was treated with Intravenous immunoglobulin. This case of Ross syndrome is reported for its rarity as well as a clue to its probable autoimmune origin and treatment option with intravenous immunoglobulins.

## Introduction

Ross syndrome is an uncommon disorder characterized by the triad of segmental anhidrosis, hyporeflexia, and tonic pupils.[1] While tonic pupil and reduced sweating can be attributed to the affection of postganglionic cholinergic fibres projecting to the iris and sweat glands, the pathogenesis of diminished or lost tendon jerks remains obscure.[2] Evidence of other autonomic dysfunctions in relation to this syndrome is very infrequent including cardiovascular dysfunction, diarrhea and coughing.[3] Only a few such cases have been reported in the literature.[4] We report a case who in addition had ANA positivity and was treated with intravenous immunoglobulin (IVIg).

## Case Report

A 28-year-old serving soldier presented with complaints of loss of sweating on his right forearm since September 2003 while posted at Leh (Ladakh). He progressively developed such anhidrotic areas on his left leg, back and feet over the next few months with associated burning sensation during summers. He also had dryness and fissuring of both hands and feet. Since the past one year he noticed visual impairment in that he could see distant objects better with his right eye and closer objects better with left with associated headache while trying to see nearer objects. There was no history suggestive of orthostatic hypotension, impotence, bowel or bladder difficulties. There was no history of burns, injury to spine or symptoms suggestive of connective tissue disorder. There was no family history of similar problems.

**Figure 1:**
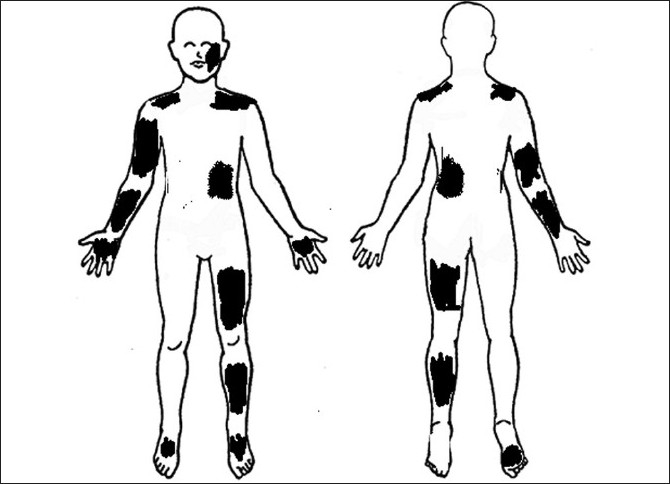
Areas of anhidrosis

On examination, anhidrosis was present in patches over face, both shoulders, right arm and forearm, left side of back, both palms, left thigh, left leg and right sole [Figure 1]. The unaffected areas had compensatory hyperhidrosis. Blood pressure measurements and head up tilt test did not show postural hypotension. Ankle jerk was absent bilaterally. Other deep tendon reflexes were normal.

**Figure 2:**
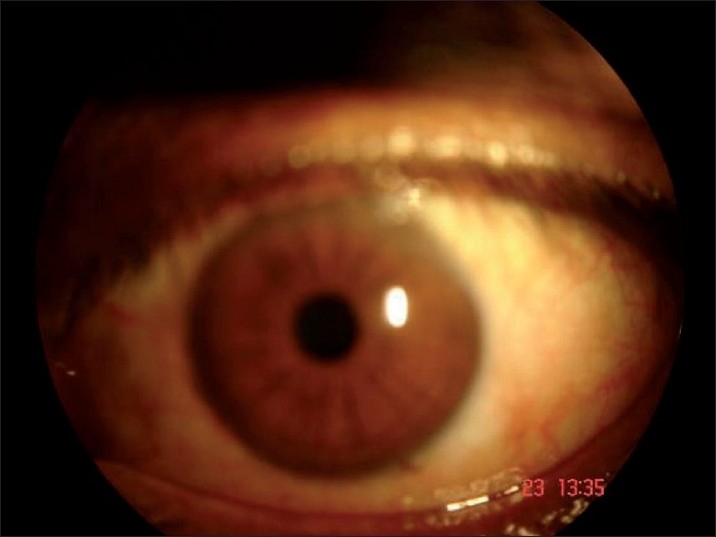
Smaller left pupil

**Figure 3:**
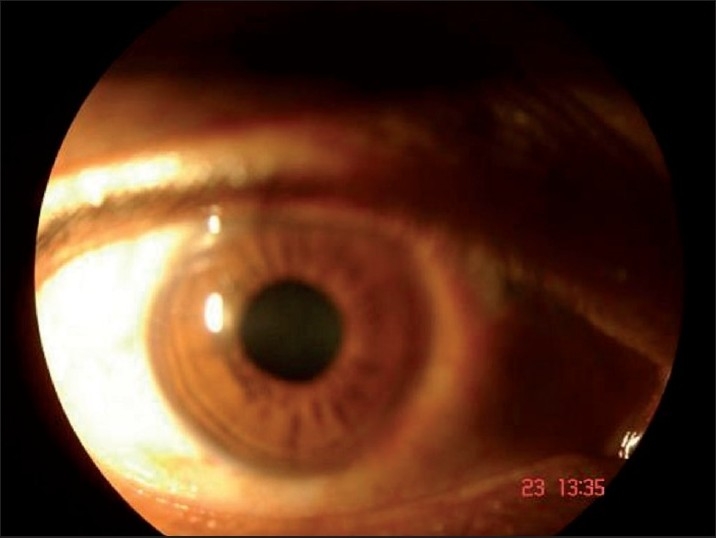
Larger tonic right pupil

**Figure 4:**
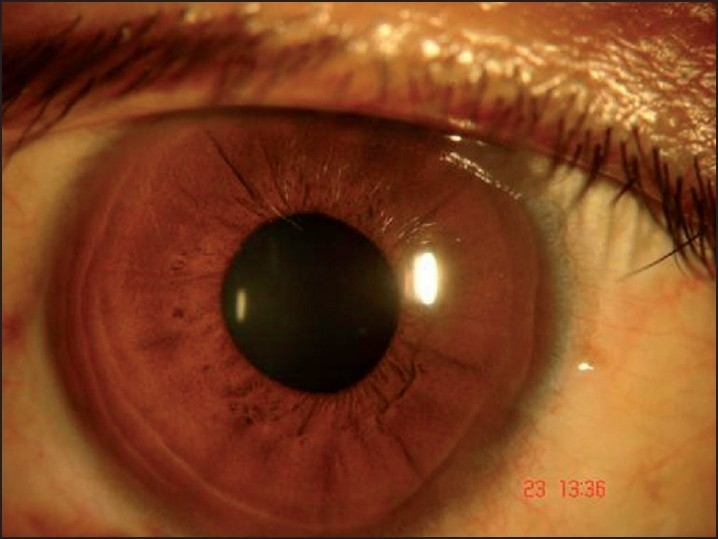
Segmental reaction in right eye

**Figure 5:**
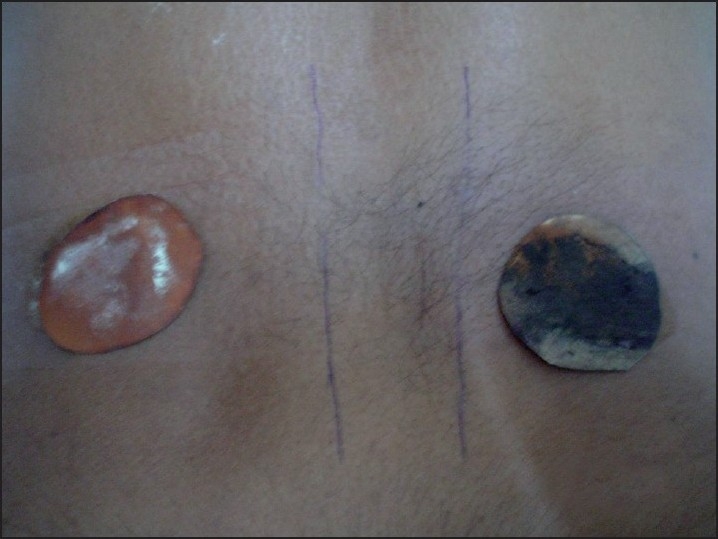
Starch Iodide test confirming anhidrosis on right side back

Ophthalmological examination revealed a best-corrected visual acuity of 20/20 in left eye and 20/36 in right eye. Torch light examination revealed anisocoria with the right pupil larger in size than the left [Figures 2 and 3]. A detailed evaluation of pupil on slit lamp revealed right-sided tonic pupil. Segmental reaction of pupillary sphincter to light was also evident [Figure 4]. There was light near dissociation. Presence of tonic pupil was confirmed by increased sensitivity and response to diluted pilocarpine eye drops. Rest of anterior segment and fundus were normal. Other systems were essentially normal. Sweat test done using the starch iodide method revealed anhidrosis in the patches mentioned above [Figure 5]. Intradermal pilocarpine 1:10,000 failed to elicit sweating in the anhidrotic areas.

Laboratory evaluation including complete blood cell count, liver, renal and thyroid function tests, blood sugar and serum electrolytes were normal. ANA was repeatedly positive while dsDNA was negative. VDRL was non-reactor. ECG, EEG and NCV were within normal limits. X ray and MRI Scan of the cervical spine were normal. Skin biopsy revealed normal sweat glands.

Since patient showed ANA positivity and the probable etiology being autoimmune, IVIg was administered in a total dose of 2g/kg given as 20 gm/day for 5 days. There was a two- fold fall in ANA titer from the initial 1:80 on investigation after 3 months. The patient has been observed for 4 months following therapy. There has been no further progression of the disease process. Topical aluminium hydrate powder was given for the hyperhidrotic areas with minimum relief.

## Discussion

Anhidrosis may be observed in a wide variety of neurological and dermatological disorders. Segmental anhidrosis has been reported in Shy-Drager disease, multiple sclerosis, diabetes mellitus, leprosy and polyneuropathies.[5] The Holmie-Adie syndrome (HAS) comprises of tonic pupil and absent stretch reflexes. The tonic pupil is large, has poor light reflexes and delayed pupillary contraction on near vision with even further delayed pupillary redilatation. There is greater impairment of reaction to light than accommodation, which is explained by the 30-fold excess of neurons to the ciliary body compared with those to the iris sphincter. The tonic pupil is initially monolateral, but frequently progresses to involve the other eye. With time a partial recovery of accommodation may occur. In about 10% cases there is a permanent failure of the pupil to react to light or near vision. The absence of deep tendon reflexes is characteristic of HAS with loss of the Achilles tendon reflex being most frequent. Once areflexia is established, it is permanent.[6]

The association of the HAS syndrome and hypohidrosis, namely the Ross syndrome was first described by Ross in 1958.[7] Injury to postganglionic cholinergic fibers is believed to account for the tonic pupil and sweating disturbance respectively.[8] Areflexia is the result of impaired spinal monosynaptic connections. The relationship between the times of onset of the pupillotonia, hypoactive deep tendon reflexes and hypohidrosis is variable.

In our case, all three components of the triad of Ross syndrome namely tonic pupil, patchy anhidrosis and areflexia were present. Anhidrosis preceded tonic pupils by 3 years. Intradermal pilocarpine failed to elicit sweating in the anhidrotic areas confirming a postganglionic defect.

The exact cause of this degenerative disorder of postganglionic cholinergic fibres is not known. Sawhney *et al*.[9] found associated congenital anomalies of the spine in the areas affected and suggested a developmental origin. However we found ANA positivity in our patient, which may suggest an autoimmune origin. This has not been reported in literature earlier. It has been recently reported that HAS which is closely related to Ross syndrome is associated with celiac disease and autoimmune hepatitis. This association suggests a common immunological background for these entities.[10] 3 cases of HAS have been attributed to autoimmunity in literature.[11] Autoantibodies against ganglionic nicotinic receptors have been found to induce autonomic neuropathy in certain reports, similar to the pathophysiology of myasthenia gravis.[12,13] Harlequin syndrome also related to Ross syndrome is found to be probably caused by an autoimmune inflammatory mechanism against upper cervical sympathetic ganglia.[14] All these factors suggest that Ross syndrome could also be autoimmune in origin.

No treatment for this progressive disorder has yet been discovered. Treatments for the compensatory hyperhidrosis including Botulinum toxin and tap water iontophoresis have been tried with variable results. Our patient was given IVIg for duration of five days. The disease process has not progressed further after a review period of 4 months. ANA titre at the time of presentation was 1:80. ANA was repeated after therapy. There was a two-fold fall but probably IVIg needs to be given in more cases to assess the relation of ANA titer to therapy. IVIg has been used with considerable success in various autoimmune disesase, including autoimmune neuropathies. Since Ross Syndrome is probably autoimmune in origin and autoantibodies may be involved, IVIg was given in the hope of blocking the autoantibodies directly, or antagonise their action on neuro-receptors. ANA positivity added to the possibility of autoantibodies being involved in the pathogenesis and thus enhanced the indication for IVIg. Further trials however need to be done to ascertain the role of autoimmunity and IVIg in Ross syndrome. This case has been reported for its rarity, a clue to its probable autoimmune origin and hence a treatment option of IVIg which may pave way for its ultimate treatment.
